# Influenza A virus nucleoprotein derived from *Escherichia coli* or recombinant vaccinia (Tiantan) virus elicits robust cross-protection in mice

**DOI:** 10.1186/1743-422X-9-322

**Published:** 2012-12-29

**Authors:** Baoying Huang, Wenling Wang, Renqing Li, Xiuping Wang, Tao Jiang, Xiangrong Qi, Yingying Gao, Wenjie Tan, Li Ruan

**Affiliations:** 1 Biotech Center for Viral Disease Emergency, National Institute for Viral Disease Control and Prevention (IVDC), Chinese Center for Disease Control and Prevention (CCDC), Changbai Road 155, Changping District, Beijing, 102206, People’s Republic of China

## Abstract

**Background:**

Immunity to conserved viral antigens is an attractive approach to develop a universal vaccine against epidemic and pandemic influenza. A nucleoprotein (NP)-based vaccine has been explored and preliminary studies have shown promise. However, no study has explored the immunity and cross-protective efficacy of recombinant NP derived from *Escherichia coli* compared with recombinant vaccinia virus (Tiantan).

**Methods:**

Recombinant NP protein (rNP) from influenza virus A/Jingke/30/95(H3N2) was obtained from *E. coli* and recombinant vaccinia virus (Tiantan) RVJ1175NP. Purified rNP without adjuvant and RVJ1175NP were used to immunize BALB/c mice intramuscularly. Humoral immune responses were detected by ELISA, while cell-mediated immune responses were measured by *ex vivo* IFN-γ ELISPOT and *in vivo* cytotoxicity assays. The cross-protective efficacy was assessed by a challenge with a heterosubtype of influenza virus A/PR/8/34(H1N1).

**Results:**

Our results demonstrate that a high dose (90 μg) of rNP induced NP-specific antibodies and T cell responses that were comparable with those of RVJ1175NP in mice. Importantly, the survival ratio (36, 73, and 78%) of the vaccinated mice after the influenza virus A/PR/8/34(H1N1) challenge was rNP vaccine dose-dependent (10, 30, and 90 μg, respectively), and no significant differences were observed between the rNP- and RVJ1175NP-immunized (91%) mice.

**Conclusions:**

Influenza A virus NP derived from *E. coli* or recombinant vaccinia (Tiantan) virus elicited cross-protection against influenza virus in mice, and the immune response and protective efficacy of rNP were comparable to RVJ1175NP. These data provide a basis for the use of prokaryotically expressed NP as a candidate universal influenza vaccine.

## Background

Influenza virus causes a highly contagious and acute respiratory disease [1]. Vaccination is the primary strategy for preventing and controlling epidemic and pandemic influenza [2,3]. Currently, licensed influenza vaccines are trivalent live attenuated or inactivated killed virus vaccines, consisting of three strains of each virus (influenza A H1N1 and H3N2 and one influenza B) thought to be most prevalent in the upcoming influenza season [4,5]. However, these vaccines elicit neutralizing antibodies against the highly variable hemagglutinin (HA) of influenza virus, providing protection against homologous but non-antigenically distinct heterologous viruses. Thus, these vaccines must be frequently reformulated to match the circulating strains [6,7]. In addition, current commercial influenza vaccines are produced by propagating the virus in embryonated chicken eggs, which is time-consuming and requires one egg per vaccine dose [8,9]. Therefore, the development of a vaccine that induces cross-protection against variant subtypes of influenza A virus and which can be produced quickly at high quantities is desirable.

The highly conserved nucleoprotein (NP) of influenza A virus is an attractive candidate for a broad-spectrum influenza vaccine [10-13]. NP could generate subtype cross-reactive cytotoxic T lymphocyte (CTL) immunity to accelerate viral clearance in mice and humans [14,15], and the non-neutralization antibodies induced by NP play a role in heterosubtypic immunity in mice [16,17]. Previous studies have demonstrated that NP induces heterosubtypic protection when used as a vaccine component. NP-based vaccines, including DNA vaccines [18,19], viral vector vaccines [20-22], peptide vaccines [23], protein subunit vaccines [24,25], and multi-antigenic vaccines [26-28], can generate cross-protection. Recently, a phase I clinical trial was conducted in healthy adults using a modified vaccinia virus Ankara (MVA) vector expressing influenza NP and matrix protein 1 (MVA-NP+M1). In that study, a challenge with influenza H3N2 and H1N1 showed that the MVA-NP+M1 vaccine was safe and immunogenic in humans [29,30]. We previously constructed a recombinant vaccinia virus (Tiantan) RVJ1175NP expressing the NP of influenza virus A/Jingke/30/95(H3N2), which elicited significant protective efficacy in mice [20]. However, the production of this viral vector vaccine was complicated, and the pre-existing vector antibody may interfere with vaccination efficacy. Thus, it is important to identify a convenient method for large-scale NP production that does not require embryonated eggs or cell culture.

*Escherichia coli* expression systems can facilitate the rapid and economical production of recombinant proteins [31,32]. The expression and purification of a single antigenic protein in bacterial culture may be a simple and rapid strategy for generating large quantities of influenza vaccine [33-36]. However, few studies of the immunogenicity and protective efficacy of recombinant NP expressed in *E. coli* have been performed, and no investigation has compared the efficacy of NP from prokaryotic expression systems with eukaryotic expression systems. To determine whether *E. coli*-expressed NP could be used as a broad-spectrum influenza vaccine, a comparison of the immunogenicity and protective efficacy of prokaryotic- and eukaryotic-expressed NP is required.

In this study, we purified recombinant NP (rNP) from influenza virus A/Jingke/30/95(H3N2) expressed in *E. coli*, and constructed a recombinant vaccinia virus (Tiantan) RVJ1175NP expressing the same NP. The immunogenicity and cross-protective efficacy of the rNP was compared with that of RVJ1175NP in BALB/c mice. We found that the *E. coli*-expressed rNP induced NP-specific antibodies and a T cell response at high doses. Additionally, the cross-protective efficacies of the rNP against a lethal challenge with heterosubtype influenza virus A/PR/8/34(H1N1) were comparable to those of RVJ1175NP. These data provide a basis for the use of *E. coli*-expressed NP as a potential universal influenza vaccine.

## Results

### Characterization of rNP purified from *E. coli* or RVJ1175NP

**Figure 1:**
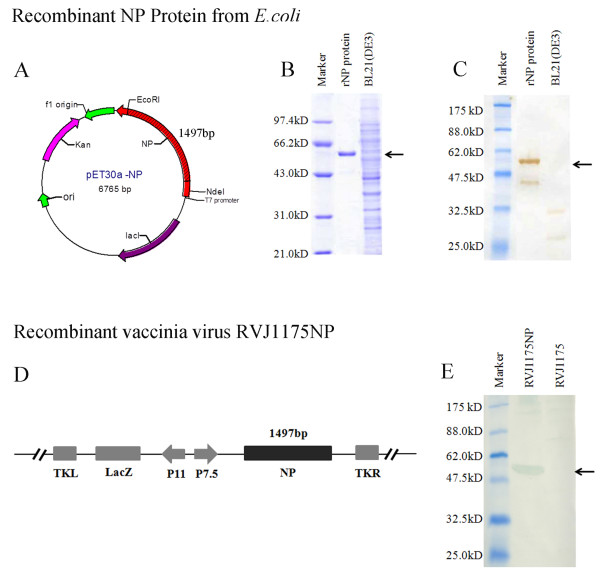
**Characterization of rNP purified from *E. coli *transformed with pET30a-NP or expressed from BHK cells infected with RVJ1175NP. (A)** Schematic representation of the expression plasmid (pET30a-NP) used to express the NP of influenza A/Jingke/30/95(H3N2) in *E. coli* BL21(DE3). **(B)** SDS-PAGE of purified rNP. Purified rNP was fractionated by 10% SDS-PAGE and stained with Coomassie blue. BL21(DE3) transformed with pET-30a was used as a negative control. **(C)** Western blot analysis of purified rNP with mouse polyclonal antibodies specific for NP. **(D)** Schematic representation of RVJ1175NP encoding the influenza NP gene. ITR, inverted terminal repeat; P7.5 K, P7.5 later promoter; P11K, P11 later promoter; TKR, right thymidine kinase; TKL, left thymidine kinase. **(E)** Western blot analysis of influenza NP expressed in BHK cells infected with RVJ1175NP using mouse polyclonal antibodies specific for NP. BHK cells infected with RVJ1175 were used as a negative control. The expression bands for NP and their molecular weights are indicated.

To assess the efficacy of rNP expressed in *E. coli* as a candidate universal influenza vaccine, we constructed an expression plasmid, pET30a-NP, to express rNP of influenza A/Jingke/30/95(H3N2) in *E. coli* BL21(DE3) (Figure 1A), as well as a recombinant vaccinia virus RVJ1175NP expressing NP (Figure 1D).

The NP gene of influenza A/Jingke/30/95(H3N2) was optimized and cloned into pET30a for expression in BL21(DE3) (Figure 1A). Untagged soluble recombinant protein was purified using ion exchange and size exclusion chromatography. SDS-PAGE demonstrated that the rNP was >90% pure (Figure 1B). The presence of purified rNP was confirmed by Western blot analysis with mouse anti-NP polyclonal antibodies (55 kDa), whereas the control BL21(ED3) did not produce NP protein (Figure 1C). BHK cells infected with RVJ1175NP or RVJ1175 were analyzed by Western blotting with the same mouse anti-NP polyclonal antibodies (Figure 1E). Our results showed that RVJ1175NP expressed the proteins as expected (55 kDa), whereas the control RVJ1175 did not produce the protein. The above results demonstrate that NP was successfully expressed and purified.

### Comparable NP-specific antibody responses were induced in both rNP- and RVJ1175NP-immunized mice

**Figure 2:**
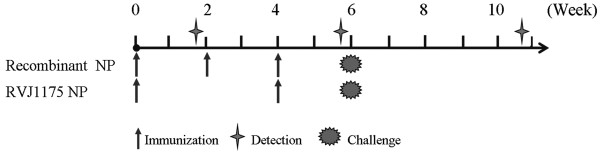
**Immunization schedule for the BALB/c mice.** Mice were immunized i.m. three times, two weeks apart with 10, 30, or 90 μg of rNP or PBS alone, respectively. Age- and sex-matched mice were immunized twice, four weeks apart with 2×10^7^ PFU per mouse of RVJ1175NP as a positive control. Serum samples from four mice were collected ten days after each immunization. Ten days after the last immunization, five mice in each group were sacrificed for *ex vivo* IFN-γ ELISPOT and *in vivo* cytotoxicity assays. Next, eleven mice in each group were challenged intranasally with 10^4^ TCID_50_ (10×MLD_50_) of influenza A/PR/8/34(H1N1) ten days after the last immunization.

The immunization schedule is shown in Figure 2 and Table 1. To analyze NP-specific humoral immunity, serum samples from four mice per group were collected ten days after each priming and boosting event, and purified NP was used to coat a 96-well plate to detect NP-specific IgG antibodies.

**Table 1:** Immunization program

**Group**	**Immunogen**	**Dose**	**Number of mice**	**Immunization route**
1	PBS	0	30	i.m.
2	rNP	10 μg	30	i.m.
3	rNP	30 μg	30	i.m.
4	rNP	90 μg	30	i.m.
5	RVJ1175NP	2×10^7^ PFU	30	i.m.

Note: Amino acid sequences of NP derived from A/Jingke/30/95(H3N2).

**Figure 3:**
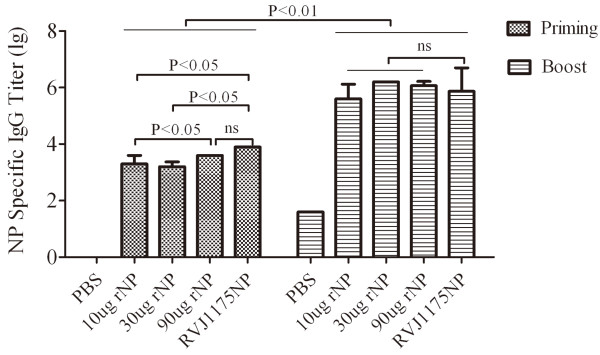
**Comparable NP-specific antibody responses were induced in both the rNP- and RVJ1175NP-immunized mice.** BALB/c mice were immunized i.m. with 10, 30, or 90 μg of rNP or PBS alone, three times, two weeks apart. Mice immunized with RVJ1175NP (2×10^7^ PFU) twice, four weeks apart, were used as positive controls. Serum samples were collected ten days after priming and boost immunization. NP-specific IgG responses were measured by ELISA as described. All data are shown as the log10 geometric mean titer ± standard deviation of four mice from each group.

As shown in Figure 3, after the priming immunization, the NP-specific antibody titer in each rNP-vaccinated group was slightly lower than that in the RVJ1175NP-vaccinated group (2×10^3^-4×10^3^ vs. 8×10^3^). Statistically significant differences were observed between 10 μg NP-, 30 μg NP-, and RVJ1175NP-vaccinated groups (P<0.05), as well as between the 10 and 90 μg NP-vaccinated groups (P<0.05). No statistically significant differences were observed between the 90 μg NP- and RVJ1175NP-vaccinated groups. However, after boosting immunization, the NP-specific antibody titer increased markedly (2×10^3^-4×10^3^ vs. 4×10^5^-1×10^6^, P<0.01), and no significant differences were found between the rNP-vaccinated groups, or between the rNP- and RVJ1175NP-vaccinated groups. The NP-specific antibody titer in each rNP-vaccinated group was slightly higher than that in the RVJ1175NP-vaccinated group (4×10^5^-1×10^6^ vs. 3×10^5^).

These results indicate that rNP elicited comparable NP-specific humoral immunity to RVJ1175NP.

### Comparable moderate T cell immune responses were induced in high-dose rNP- and RVJ1175NP-immunized mice

To detect NP-specific T cell-mediated immune responses, five mice from each group were sacrificed ten days after the last immunization, and specific cellular immune response against the NP_147-155_ epitope were detected by *ex vivo* IFN-γ ELISPOT and *in vivo* cytotoxicity assays, as described.

**Figure 4:**
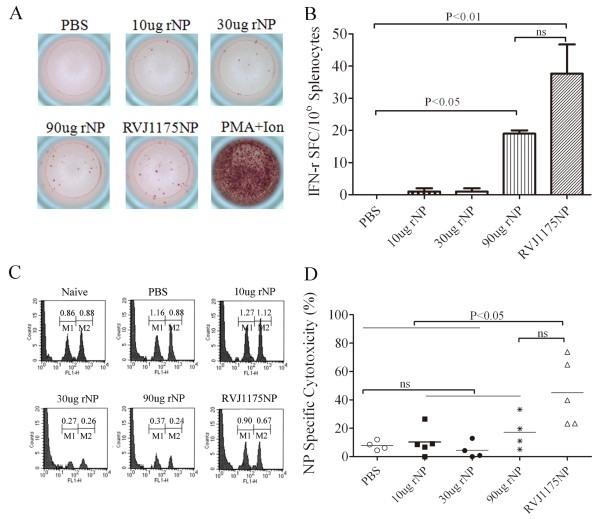
**Comparable moderate T cell immune responses were induced in both high-dose rNP- and RVJ1175NP-immunized mice.** BALB/c mice were immunized i.m. with 10, 30, or 90 μg of rNP or PBS alone, three times, two weeks apart. Mice immunized with RVJ1175NP (2×10^7^ PFU) twice, four weeks apart, were used as a positive control. Ten days after the last immunization, mice were sacrificed for *ex vivo* IFN-γ ELISPOT and *in vivo* cytotoxicity assays, as described. All data are shown as the mean ± standard deviation for two independent experiments. **(A)** Specific T cell responses were tested by *ex vivo* IFN-γ ELISPOT assays against NP_147-155_ in mice (n = 4 per group). Splenocytes (500,000) were incubated *ex vivo* with or without 5 μg/ml NP_147-155_. The plates were then incubated for 20-24 h. The left panel shows the number of IFN-γ-producing cells as determined by ELISPOT. Each histogram is representative of data from one mouse; a portion of the results are presented here. **(B)***In vivo* cytotoxicity assays to determine the specific lytic potential of effector CD8^+^ T cells in mice. Target cells were transferred to immunized or naïve mice (n = 5 per group). The left histograms show the number of CFSE_low_ unpulsed (M1) or the CFSE_high_ NP_147-155_-pulsed (M2) mouse spleen cells. Each histogram is representative of data from one mouse; a portion of the results are presented here. The right graph indicates the percentage of specific killing for NP_147-155_-pulsed targets from the indicated groups.

To identify IFN-γ-positive SFC against the NP_147-155_ epitope, we performed *ex vivo* IFN-γ ELISPOT assays. As indicated in Figure 4A, compared with the PBS control group (<5 SFC/10^6^ splenocytes), no significant IFN-γ-positive SFC against the NP_147-155_ epitope were detected in the 10 or 30 μg NP-immunized mice (<10 SFC/10^6^ splenocytes). However, a significant number of SFC were detected in both the 90 μg NP- and RVJ1175NP-vaccinated groups (19 ± 1 SFC/10^6^ splenocytes, 35 ± 1 SFC/10^6^ splenocytes, respectively), and the immune responses were significantly different when compared with PBS-immunized mice (P<0.05 and 0.01, respectively) (Figure 4A).

To assess the lytic potential of effector CD8^+^ T cells in the mice, we examined cell cytotoxicity *in vivo* by transferring target cells pulsed with NP_147-155_ into mice. The results of our *in vivo* cytotoxicity assays were in agreement with those of the *ex vivo* IFN-γ ELISPOT assays. As shown in Figure 4B, compared with 8% of the NP_147-155_ peptide-pulsed targets eliminated in PBS control mice, neither the 10 (10%) nor the 30 (4.5%) μg NP-vaccinated groups showed any marked cytotoxic effect. A cytotoxic effect was detected, however, in the 90 μg NP- (17%) and RVJ1175NP-vaccinated groups (45%). Significant differences were observed between the RVJ1175NP- and PBS-vaccinated groups (P<0.05), but not between the RVJ1175NP- and 90 μg NP-vaccinated groups. Taken together, these results indicate that a high dose of rNP elicited a weak T cell response, similar to RVJ1175NP.

### Comparable protective efficacies against a lethal challenge with heterosubtype influenza virus A/PR/8/34(H1N1) were induced in both the rNP- and RVJ1175NP-immunized mice

To assess the cross-protection provided by rNP and RVJ1175NP, we challenged the immunized mice with 10×MLD_50_ of influenza virus A/PR/8/34 (H1N1) ten days after the last immunization (eleven mice per group) and monitored their weight changes and survival ratios for three weeks.

**Figure 5:**
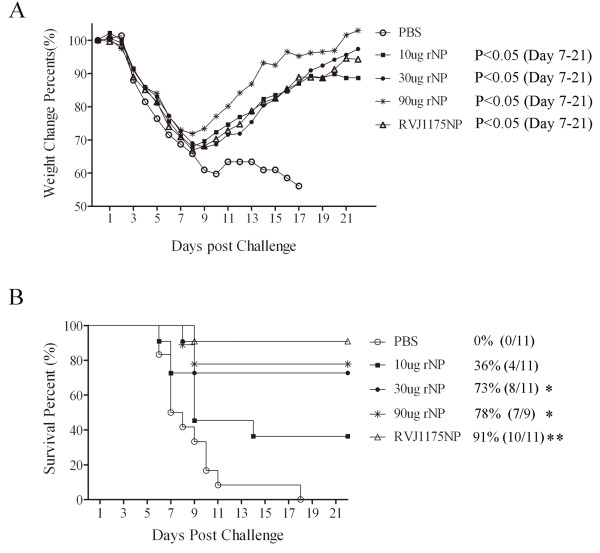
**Comparable protective efficacies against a lethal challenge by heterosubtype influenza virus A/PR/8/34(H1N1) were induced in both rNP- and RVJ1175NP-immunized mice.** Ten days after the last immunization, mice were anesthetized using a pentobarbital sodium solution and challenged intranasally with a lethal dose (10×MLD_50_) of influenza virus A/PR/8/34(H1N1). Weight changes and the survival of the mice were monitored for three weeks (n = 11 per group, except n = 9 for the 90 μg rNP group, as two mice were killed while being anesthetized). **(A)** Weight changes after the viral challenge. The average percent of the initial weight is expressed as a percentage of the examined day relative to the weight prior to the challenge. **(B)** Percent of mice surviving after the challenge. Survival was analyzed using the Kaplan-Meier log-rank test. Significant differences in survival were compared with the PBS control groups (*, P<0.05; **, P<0.01).

The observed weight changes are shown in Figure 5A. Body weight decreased to the lowest level in all groups at days 8-9 after the influenza virus A/PR/8/34(H1N1) challenge. Subsequently, body weight decreased continuously in the PBS group until day 17 when the last mouse died. In the RVJ1175NP- and each rNP-vaccinated group, the body weights of the mice returned to baseline at day 9. The body weights were restored to baseline most rapidly in the 90 μg NP-immunized group. The body weights were completely restored in each group of surviving mice at day 21. Statistically significant differences in weight loss were observed between the rNP- and PBS-immunized groups on days 7-21 (P<0.05), between the RVJ1175NP- and PBS-immunized groups, and between the 90 μg NP and other vaccinated mice on days 7-21 (P<0.05). No significant differences were observed between the 10 μg NP-, 30 μg NP-, and RVJ1175NP-vaccinated mice.

The survival ratios are shown in Figure 5B and Table 2. Compared with the PBS group (0%, 0/11), the survival ratio in the RVJ1175NP group was 91% (10/11, P=0.0001), while the survival ratios in the rNP group at doses of 10, 30, and 90 μg were 36 (4/11, P=0.09), 73 (8/11, P=0.01), and 78% (7/9, P=0.005), respectively. Excluding the 10 μg NP group, statistically significant differences in the survival ratios were observed between the rest of the rNP- and PBS-vaccinated mice (P≤0.01). No significant differences were observed between the rNP- and RVJ1175NP-vaccinated mice.

**Table 2:** Mouse survival calculation after influenza virus A/PR/8/34(H1N1) challenge

**Immunogen**	**Number surviving/total**	**Survival ratio (%)**	**P (versus PBS)**
PBS	0/11	0	/
10 μg of rNP	4/11	36.4	0.09
30 μg of rNP	8/11	72.7	0.01
90 μg of rNP	7/9	77.8	0.005
RVJ1175NP	10/11	91.0	0.0001

Note: The immunized mice were challenged with 10×MLD_50_ of influenza virus A/PR/8/34(H1N1) ten days after the last immunization. N = 11 per group, except n = 9 for the 90 μg rNP group because two mice were killed while being anesthetized. No significant differences were observed between each rNP group, or between the rNP- and RVJ1175NP-immunized groups. The P-values shown here were calculated compared to the PBS control group.

The above results indicate that rNP elicited comparable cross-protection to RVJ1175NP in mice, and the survival ratios tended to increase with a higher dose of rNP.

### An influenza virus A/PR/8/34(H1N1) challenge boosted NP-specific immunity and increased mouse survival

To explore the possible mechanisms of protection, 35 days after influenza virus A/PR/8/34(H1N1) challenge, humoral and cell-mediated immune responses were detected in the surviving mice. An examination of the PBS group was not possible as none of the mice survived.

**Figure 6:**
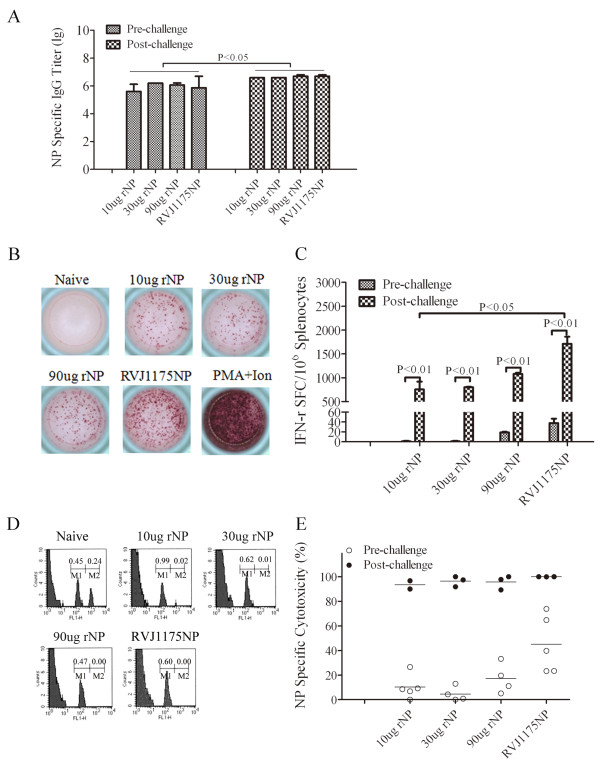
**An influenza virus A/PR/8/34(H1N1) challenge boosted the NP-specific immune responses in the surviving mice.** To explore the possible mechanisms of immunological protection, 35 days after the challenge with influenza virus A/PR/8/34(H1N1), humoral and cell-mediated immune response changes were explored. The analysis was performed for the surviving mice; no analysis was performed for the mice in the PBS group since none survived. **(A)** NP-specific IgG antibody titers were tested by ELISA in the surviving mice. **(B)** Specific T cell responses were tested directly by *ex vivo* IFN-γ ELISPOT assays against NP_147-155_ in the surviving mice. **(C)***In vivo* cytotoxicity assays were used to determine the specific lytic potential of effector CD8^+^ T cells in the surviving mice. The results are representative of two experiments (n = 2-4 per experiment).

Based on our ELISA results (Figure 6A), NP-specific humoral immune responses were slightly increased in the surviving mice (P<0.05). The NP-specific IgG titer increased from pre-challenge levels of 4×10^5^-1×10^6^ to 3×10^6^-5×10^6^ in each immunized group. No significant differences were found between these groups. The strength of the humoral immune response in the rNP groups was similar to that in the RVJ1175NP group.

Based on our *ex vivo* IFN-γ ELISPOT assays (Figure 6B), the number of IFN-γ-positive SFC against NP_147-155_ was markedly increased in the surviving mice (P<0.01). Compared with pre-challenge (<50 SFC/10^6^ splenocytes), the average number of SFC was 756, 802, 1712, and 1080 SFC/10^6^ splenocytes in the 10, 30, and 90 μg rNP group and in the RVJ1175NP group, respectively. The number of SFC increased significantly (P<0.01), and tended to increase with higher immunization doses. Significant differences were observed only between the 10 μg NP- and RVJ1175NP-vaccinated surviving mice (P<0.05), and differences were not observed between the rNP-immunized surviving mice at different doses, nor between the rest of the rNP groups and the RVJ1175NP-immunized groups.

As revealed by our *in vivo* cytotoxicity assays (Figure 6C), NP_147-155_ peptide-pulsed target killing was increased markedly in the surviving mice (P<0.01). Compared with the pre-challenged mice (30%), the cytotoxic rates were increased to 93, 96, and 96% with 10, 30, and 90 μg of rNP in the surviving mice, respectively, and increased cytotoxic effects were observed in the surviving RVJ1175NP mice (from 45 to 100% ). No differences were observed between each group.

The above results indicate that a challenge with influenza virus increased NP-specific immune responses in the surviving mice, especially NP-specific cell-mediated immune responses.

## Discussion

Influenza is a major cause of morbidity and mortality worldwide. Vaccination is the most effective strategy to control influenza epidemic and pandemics. However, currently licensed influenza vaccines are annual vaccines that induce subtype-specific virus-neutralizing antibodies against the highly variable surface antigen HA; thus, they do not protect against new subtypes or antigenic variants. In addition, conventional egg-dependent manufacturing is time-consuming and expensive. Therefore, it is important to produce a vaccine that induces cross-protection and which can be produced rapidly and inexpensively. Immunity to conserved NP antigens is an attractive approach for developing universal influenza vaccines, and a subunit vaccine based on a prokaryotic production system could be rapidly and inexpensively produced. Although many studies have explored the protective capacity of NP as a component of a DNA vaccine or expressed by viral vectors, it remains unclear whether similar results could be obtained with rNP. In this study, we compared the immunogenicity and cross-protection of rNP from *E. coli* with recombinant vaccinia (Tiantan) virus RVJ1175NP in mice. Our results demonstrate that a high dose of rNP induced comparable anti-NP antibody and T cell responses to RVJ1175NP in mice. Importantly, the protective efficacies of rNP were comparable with that of RVJ1175NP. These data provide a basis for the use of *E. coli*-expressed NP as a candidate universal influenza vaccine for further study.

A wide variety of NP-based vaccine formulations have been evaluated for cross-protection from this highly conserved antigen [18-30]. Recombinant vaccinia viruses are conventionally used to study the immunogenicity of foreign proteins [37-42]. The vaccinia virus Tiantan strain was used as a vaccine against smallpox in China before 1980, and it is now widely used as a vector [43,44]. We previously used RVJ1175 expressing the potential cross-protective antigens of NP, Matrix protein 1 (M1) and Polymerase basic 1 (PB1) of influenza A/Jingke/30/95(H3N2) with the vaccinia virus Tiantan strain to induce cross-protection in Balb/C mice, the results indicated that NP is the most effective antigen among the antigens we tested, and the survival rate of the RVJ1175NP immunized mice could achieved as high as almost 100% against the lethal challenge of influenza influenza virus A/PR/8/34 (H1N1) with a challenge dose ranged from 1LD50 to 5LD50[20]. However, the complicated production process and pre-existing vector immunity may interfere with the vaccine. *Escherichia coli*-based expression systems are the simplest and fastest way to generate large quantities of influenza vaccine. However, no investigation has compared the immunity and cross-protective efficacy of rNP derived from *E. coli* with recombinant vaccinia virus (Tiantan). To explore the potential of *E. coli*-expressed NP as a candidate universal influenza vaccine, we compared the immunogenicity and efficacy of three doses of rNP (10, 30, and 90 μg) from *E. coli* with that of RVJ1175NP.

Antibodies against NP are non-neutralizing, and although viral infection and some replication continues to occur, it can limit viral replication and reduce illness severity [16,17,45,46]. In the present study, the results of NP-specific IgG detection demonstrated that the *E. coli*-expressed NP without adjuvant could elicit a strong humoral immune response, similar to RVJ1175NP. Recently, Lamere et al. [45] demonstrated that systemic immunization with NP accelerated the clearance of a 2009 pandemic H1N1 influenza virus isolate in an antibody-dependent manner, and that anti-NP IgG specifically promoted influenza virus clearance in mice through a mechanism involving both FcRs and CD8^+^ T cells [46]. These studies strongly suggest that antibodies induced by immunization with NP can be used to elicit cross-protection.

Currently, NP is thought to play a role in protection mainly through the CTL cross-reaction. Several previous studies have confirmed that NP-based vaccines inducing cell-mediated immunity can provide cross-protection against a heterosubtypic influenza virus challenge [14,15,18-28]. In this study, cell-mediated immune responses were assessed by measuring *ex vivo* IFN-γ secretion in splenocytes and *in vivo* cytotoxicity against the CD8^+^ T cell epitope NP_147-155_. Although few *ex vivo* IFN-γ-positive SFC and weak *in vivo* cytotoxicity were induced at low doses (10 or 30 μg) of NP, a weak cellular immune response could be detected at high doses (90 μg) of NP. Such T cell-mediated immunity was comparable with that induced by RVJ1175NP. These results indicate that the weak cellular immune response was boosted by increasing the immunization dose or by using an adjuvant. Additionally, although the NP-specific CD8^+^ T cell immunity of NP was weak in the present study, it might establish long-term memory cells. Therefore, a later time point should be investigated.

**Figure 7:**
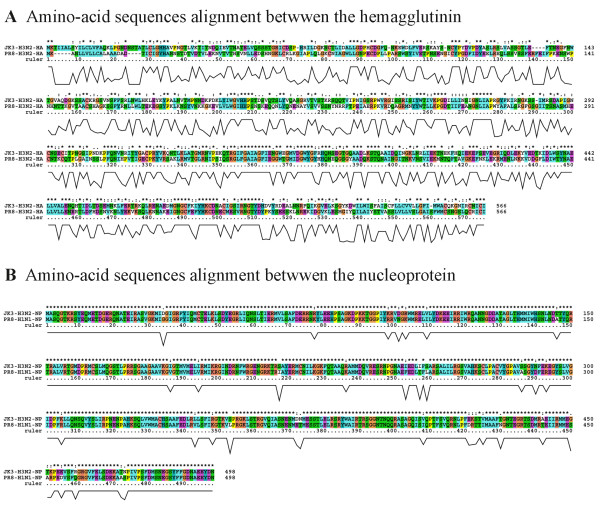
The amino-acid sequence alignment between the HA and NP of the influenza virus strains of A/Jingke/30/95 (H3N2) and A/PR/8/34 (H1N1).

To assess the cross-protection effect provided by rNP and RVJ1175NP, 10×MLD_50_ of influenza virus A/PR/8/34(H1N1) was used to challenge the immunized mice. It should be noted that, according to the amino-acid sequence alignment, the differences in amino-acid sequence between the influenza virus strains of A/Jingke/30/95 (H3N2) and A/PR/8/34 (H1N1) is 40.7% for HA and 92.6% for NP respectively (Data was analyzed by the software of Clustal X (1.8), detailed information would be seen in Figure 7 in the section of supporting file). The observed weight changes were comparable between the rNP-vaccinated mice and the RVJ1175NP-vaccinated mice. Notably, mice immunized with 90 μg of NP showed the earliest and best recovery (better than RVJ1175NP). These results indicate that rNP effectively relieved the symptoms of influenza and reduced the disease severity. In addition, the survival ratios (36, 73, and 78%) were rNP vaccine dose-dependent (10, 30, and 90 μg, respectively), and no statistical differences were observed between the rNP-immunized mice and RVJ1175NP (91%). Consistent with previous studies, the survival protective efficacy in our study was in accordance with the strength of the pre-challenge immune response [47,48]. The above results indicate that *E. coli*-expressed NP elicited cross-protection in the mice, similar to RVJ1175NP, and that the efficacy was correlated with the magnitude of the immunological response.

To explore the possible mechanisms of protection, immune responses were evaluated in the surviving mice. Compared with pre-challenge, both the NP-specific humoral and cell-mediated immune responses were increased in the surviving mice. These results suggest that NP-specific humoral and cell-mediated immune responses are closely correlated with protection. They also suggest that the protection efficacy might be further enhanced by an outbreak of seasonal influenza [49-51]. However, further support of this hypothesis is important to promote universal flu vaccine development. As under the lethal challenge of influenza virus, the examination of the PBS group was not possible as none of the mice survived, there was lack a proper control in this experiment, so further study should be designed to include an unvaccinated group infected with a sub-lethal dose of influenza virus to quantify the immune response values elicited after infection in animals without previous vaccination.

It also should be noted that, the mechanisms that rNP could induce cross-protection are complex. The general consensus favors the idea that rNP can induce CTL responses that can kill infected cells and help the host recovery from the infection [14,15,18-28], while the non-neutralizing antibodies induced by rNP contribute little to providing protective ability. However, recent studies in mice also demonstrated that antibodies against rNP also contribute to heterosubtypic immunity, and thus can limit viral replication and reduce illness severity, maybe through a mechanism involving both FcRs and CD8^+^ T cells [16,17,45,46]. In our experiment, in spite of the very low levels of CTL elicited in the rNP and RVJ1175NP vaccinated groups, survival rates were achieved as high as 73%, 78%, and 91% in the 30 μg rNP, 90 μg rNP and RVJ1175NP vaccinated group after the lethal challenge of influenza virus, respectively. The mechanism of cross-protection induced by rNP should be investigate by much more studies such as more immune response index should be examined, and passive serum transfer experiment et.al may be helpful to test the role of NP specific serum antibody in providing protection.

In addition, although previous studies have shown that mucosal immunization with NP protein could induce CTL responses, thus generate cross-protective response. And IgG produced by intraperitoneal or intramuscular injection of NP protein was not sufficient to protect mice against heterologous influenza virus, we confirmed the intramuscular injection of NP protein could provide cross-protection in Balb/C mice in this study, such protection would contribute the vaccine form we used, the immune path way of such vaccine, the remnant LPS adjuvants effect in the experiment, the immune responses type induced by the vaccine, and so on. In the future study, much more detailed information should be investigated to learn the structure of the rNP, as the rNP was in soluble form at the end of the fermentation, and the structure of the rNP may be polymer forms, thus would be more immunogenic . In addition, the antibody subtypes induced by the rNP may also be detected in future, as the IgG1, IgG2a subtype may also influence the protective efficacies of the vaccine. And more detailed indicators for the cellular immune response of NP should be investigated to learn the role of both CD8^+^ and CD4^+^ T cell immune response in the contribution of the cross-protection.

## Conclusions

In summary, our study demonstrates that the immune response and protective efficacy of rNP from *E. coli* were comparable to those of RVJ1175NP. These data provide a basis for the use of *E. coli*-expressed NP as a candidate universal influenza vaccine for further study. To the best of our knowledge, this is the first study to compare the immunity and protective efficacy of *E. coli*-expressed and vaccinia virus expressed NPs. Further work to improve the cross-protective immunity and efficacy of rNP using adjuvants or by combining it with other protective antigens, and additional influenza virus subtype challenge studies to examine the level of broad-spectrum protection, are required.

## Materials and methods

### Preparation of rNP and RVJ1175NP

The amino acid of NP protein was based on influenza virus A/Jingke/30/95(H3N2). The NP gene sequence encoding the full-lenghth of NP protein was optimized according to the codon bias of *E. coli*[52]. The recombinant NP expression vector pET30a-NP was constructed by inserting the NP gene between the *Nde*I and *Eco*RI restriction sites. Next, rNP was expressed in *E. coli* BL21(DE3) cells transformed with pET30a-NP. Briefly, bacteria were grown to log phage at cell concentration of OD600 was 0.6-0.8 in 2×YT medium with 100 mg/L kanamycin at 37°C, and protein expression was induced by adding isopropylthio-ß-D-galactoside (IPTG) to a final concentration of 0.1 mmol/L. After 10 h of further incubation at 25°C, the cells were harvested by centrifugation, and SDS-PAGE anylysis showed that rNP was mainly in the supernatants of the cell lysis, thus the rNP was expressed in soluble form in *E. coli* fermentation. Then the untagged soluble recombinant protein was purified using ion exchange exclusion chromatography by DEAE Sepharose Fast Flow column and then size exclusion chromatography by Superdex S200 column. After concentration and filter sterilization, the protein concentration was determined using a commercial bicinchonic acid (BCA) assay, and rNP concentration was 1 mg/ml in the final purified product. Endotoxin levels were determined using the Tachypleus Amebocyte Lysate assay (Chinese Horseshoe Crab Reagen Manufactory, Xiamen, China) as directed by the manufacturer, and the endotoxin level of the rNP was about 2000 EU/mg. The final purified protein was stored in PBS at -70°C until use.

The original vaccinia virus Tiantan strain and dual-promoter insertion vector pJSA1175 were produced in our laboratory [20]. The NP gene of influenza virus A/Jingke/30/95(H3N2) was inserted into the *Sma*I site of pJSA1175. Recombinant vaccinia virus was produced by the transfection of pJSA1175-NP into CEF cells that were infected with vaccinia Tiantan strain, and was designated as RVJ1175NP (Figure 1). RVJ1175NP induced marked cross-immune protection in BALB/c mice [20], and was used to immunize mice as a positive control.

### Peptide and influenza viruses

The H-2^d^ restricted class I peptide NP_147-155_ (TYQRTRALV) was synthesized commercially (Beijing Scilight Biotechnology Ltd. Co., Beijing, China). The purity of the peptide was >90% following HPLC and mass spectrum analysis. NP_147-155_ is the CTL epitope of NP in BALB/c (H-2^d^) mice and was selected as the optimal peptide by mapping influenza A/PR/8/34(H3N2) NP peptide pools [53]. The peptide was used at 5 μg/ml to analyze NP-specific T cell immune responses using *ex vivo* gamma interferon enzyme-linked immunospot (IFN-γ ELISPOT) assays and *in vivo* cytotoxicity assays. Stimulation with PMA (50 ng/ml) and ionomycin (1 μg/ml) was used as a positive control to generate and detect antigen-specific T cells by ELISPOT.

Mouse-adapted influenza virus A/PR/8/34(H1N1) was used as the challenge strain. The viruses were propagated in allantoic fluid from nine-day-old embryonated eggs at 34°C for two days. The allantoic fluid was collected, aliquoted, and stored at -70°C until use. The viral 50% lethal dose was measured in BALB/c mice (MLD_50_) and the TCID_50_ titer was detected in MDCK cells [54]. All experiments with live influenza virus were performed in a biosafety level 2 containment facility.

### SDS-PAGE and Western blotting

rNP purified from *E. coli* or expressed from RVJ1175NP was analyzed for size and purity by SDS-PAGE and Western blotting using mouse polyclonal antibodies against influenza virus NP. For the rNP, both purified protein and BL21(DE3) cell controls were lysed in SDS-PAGE sample loading buffer and then separated by SDS-PAGE, followed by staining with Coomassie brilliant blue R250. For the RVJ1175NP, BHK cells infected with RVJ1175NP or control RVJ1175 were collected after 48 h, then processed by cell lysis and separated by SDS-PAGE. For Western blot analysis, the lysates separated by SDS-PAGE were transferred by electroblotting to a polyvinylidene difluoride membrane (Millipore). The membrane was blocked for 1 h in 5% skim milk at 37°C and then incubated with polyclonal antibodies in 2% skim milk for 1 h at 37°C. After being washed three times with phosphate-buffered saline (PBS) containing 0.05% Tween-20 (PBST), the membrane was subsequently incubated in horseradish peroxidase (HRP)-conjugated secondary anti-mouse antibodies. Binding signals were visualized with 3,3,5,5- tetramethylbenzidine (TMB) as the substrate.

### Immunization and challenge

Five- to six-week-old female BALB/c mice were obtained from the Institute of Laboratory Animal Sciences, Chinese Academy of Medical Sciences and Peking Union Medical College (Beijing, China). All mouse experiments in this study followed the Regulations for Administration of Laboratory Animals of the People’s Republic of China.

The mice were immunized intramuscularly (i.m.) three times, two weeks apart with 10, 30, or 90 μg of rNP or PBS alone, respectively. Age- and sex-matched mice were immunized twice, four weeks apart with 2×10^7^ PFU per mouse of RVJ1175NP as a positive control. Blood samples were collected ten days after each immunization. Ten days after each immunization, five mice per group were sacrificed for cellular immune response assays (*ex vivo* IFN-γ ELISPOT assays or *in vivo* cytotoxicity assays). Ten days after the last immunization, eleven mice in each group were lightly anesthetized using a pentobarbital sodium solution and were challenged intranasally with 50 μl of viral suspension containing 10^4^ TCID_50_ (10×MLD_50_) of influenza A/PR/8/34(H1N1). Survival and weight loss were monitored daily for three weeks. The challenge experiment was repeated three times.

### Enzyme-linked immunosorbent assay (ELISA)

Costar 96-well plates were coated with purified NP at a concentration of 2 μg/ml at 4°C overnight. The plates were then washed with PBST and blocked with 2% bovine serum albumin (BSA) in PBS. The test samples were serially diluted in PBS containing 1% BSA and incubated at 37°C for 1 h. Diluted HRP-linked goat-anti-mouse IgG antibodies (100 μl) were added to each well, and the plates were incubated at 37°C for 1 h. TMB substrate solution (100 μl) was then added to each well. After a 5-min incubation at room temperature in the dark, the reaction was stopped by adding 50 μl of 2 M H_2_SO_4_ per well and the absorbance was measured at 450 nm. The antibody titer was defined as the reciprocal of the highest dilution that yielded an OD_450_ value ≥2.1 times of the mean value of naïve mouse serum.

### IFN-γ ELISPOT assay

The number of NP-specific IFN-γ-secreting cells in mice was counted using commercial ELISPOT assay kits (BD Biosciences) as per the manufacturer’s instructions. Briefly, anti-mouse IFN-γ monoclonal antibodies were coated on multiscreen 96-well plates at 4°C overnight. Next, the plates were washed three times and blocked for 2 h with RPMI 1640 containing 10% FBS (GIBCO) at room temperature. Spleen mononuclear cells (SMNCs) were obtained after the red cells in the spleen cell suspension were lysed. Then the freshly isolated splenocytes (5×10^5^) were transferred to each well and NP_147-155_ was added at a final concentration of 4 μg/ml. Cells without the peptide were used as a negative control and cells with PMA (50 ng/ml) and ionomycin (1 μg/ml) were used as positive controls. Following incubation for 20-24 h at 37°C in a 5% CO_2_ incubator, the cell suspensions were aspirated. All wells were washed four times with PBST, biotinylated detection antibody was added, and the plates were incubated for 2 h at room temperature. After four washes, streptavidin horseradish peroxidase antibody was added at 100 μl per well for 1 h at room temperature. Following four more washes, 100 μl of freshly prepared 3-amino-9-ethylcarbazole substrate solution was added for 15-30 min at room temperature in the dark to yield colored spots. Finally, the reaction was stopped by thoroughly rinsing with tap water. The plates were air-dried and stored in the dark until analysis. The number of spots was analyzed with a fully automated computer-assisted video image analysis system (Bioreader 4000; Bio-Sys, Karben, Germany). The average number of spot-forming cells (SFC) was adjusted to 1×10^6^ splenocytes for data display.

### *In vivo* cytotoxicity assay

An *in vivo* cytotoxicity assay was performed as described by Byers et al. [55]. Briefly, to prepare target cells for *in vivo* cytotoxic activity detection, splenocytes from naïve BALB/c mice were washed and divided into two populations. One population was pulsed with 10 μg/ml NP_1471-155_, incubated at 37°C for 4 h, and labeled with a high concentration of CFSE (10 μM) (CFSE_high_ cells). The second target population was left without peptide and was labeled with a low concentration of CFSE (1.0 μM) (CFSE_low_ cells). For intravenous injection, an equal number of cells from each population were mixed together, such that each mouse received a total of 1×10^7^ cells in 100 μl of PBS. The cells were injected into mice vaccinated previously with PBS, rNP, or RVJ1175NP. Specific *in vivo* cytotoxicity was determined by collecting the spleen from recipient mice 20 h after injection, and labeled fluorescent target cell populations were detected based on their differential CFSE fluorescence intensities by flow cytometry. Decreased numbers of CFSE_high_ cells indicated *in vivo* cytotoxicity. The percentage of specific killing was calculated as follows: Cytotoxicity = [1-(Ratio of naïve group/Ratio of experimental group)] ×100; Ratio = percentage CFSE_low_/percentage CFSE_high_.

### Statistical analysis

Statistical analyses were performed with GraphPad Prism version 5.01 (GraphPad Software, Inc., 2007) and the SPSS software package (release 12.1; SPSS Inc., Chicago, IL). Comparisons of the mean immune responses among the mouse groups were performed using analyses of variance with an unpaired *t*-test. Comparisons of antibody titers among the treatment groups were performed using Student’s *t*-test. Comparisons of the percentage of specific killing were performed with Fisher’s exact test. Comparisons of the loss of body weight and survival curves were calculated by *t*-tests and the log-rank (Mantel-Cox) test. All reported P-values were two-sided; values <0.05 were considered to be statistically significant.

### Animal ethics statement

This mouse study was conducted in strict accordance with the recommendations in the Guide for the Care and Use of Laboratory Animals of the Chinese Center for Disease control and prevention. The protocol was approved by the Committee on the Ethics of Animal Experiments of the Institute for Occupational Health and Poison Control (Permit Number: EAWE-2010-029). Serum was obtained by orbital sinus puncture. In the ELISPOT assay and *in vivo* CTL assay, mice were sacrificed by cervical dislocation. Challenge experiment was performed under sodium pentobarbital anesthesia, and all efforts were made to minimize suffering. After influenza virus challenge, mice were monitored closely for three weeks for signs of illness. Any animals in a moribund condition were euthanized.

### Supporting data file

Figure 7. The amino-acid sequence alignment between the HA and NP of the influenza virus strains of A/Jingke/30/95 (H3N2) and A/PR/8/34 (H1N1). According to the amino-acid sequences alignment by the software of Clustal X (1.8), the results demonstrated that the differences in amino-acid sequence between the influenza virus strains of A/Jingke/30/95 (H3N2) (referred as JK3 in the graph) and A/PR/8/34 (H1N1) (referred as PR8 in the graph) is 40.7% for HA and 92.6% for NP respectively. The data sets supporting the results of this article is included within the article.

## Abbreviations

BSA: Bovine serum albumin; CTL: Cytotoxic T lymphocyte; ELISA: Enzyme-linked immunosorbent assay; ELISPOT: Enzyme-linked immunospot; HA: Hemagglutinin; M1: Matrix protein 1; MLD50: 50% lethal dose in mice; NP: Nucleoprotein; PB1: Polymerase basic 1; PBS: Phosphate buffered saline; SFC: Spot Forming Cell; TCID50: 50% tissue culture infection dose.

## Competing interests

The authors declare that they have no competing interests.

## Authors’ contributions

BH and WW generated the *E. coli* expression plasmids, expressed and purified the recombinant NP protein, performed immunogenicity studies in mice, and drafted the manuscript. RL participated in generating the viral construct and detection. XW, TJ, XQ and YG participated in performing the immunogenicity studies in mice. WT participated in designing the study and edited the manuscript. LR contributed ideas to this work, directed the study, analysed and interpreted the data. All authors read and approved the manuscript.
